# *tert*-Butyl *N*-((1*S*)-2-hy­droxy-1-{*N*′-[(1*E*)-4-meth­oxy­benzyl­idene]hydrazinecarbon­yl}eth­yl)carbamate

**DOI:** 10.1107/S1600536811024263

**Published:** 2011-06-25

**Authors:** Alessandra C. Pinheiro, Marcus V. N. de Souza, Edward R. T. Tiekink, Solange M. S. V. Wardell, James L. Wardell

**Affiliations:** a Fundação Oswaldo Cruz, Instituto de Tecnologia, em Fármacos – Farmanguinhos, R. Sizenando Nabuco, 100, Manguinhos, 21041-250 Rio de Janeiro, RJ, Brazil; b Department of Chemistry, University of Malaya, 50603 Kuala Lumpur, Malaysia; c CHEMSOL, 1 Harcourt Road, Aberdeen AB15 5NY, Scotland; d Centro de Desenvolvimento Tecnológico em Saúde (CDTS), Fundação Oswaldo Cruz (FIOCRUZ), Casa Amarela, Campus de Manguinhos, Av. Brasil 4365, 21040-900 Rio de Janeiro, RJ, Brazil

## Abstract

The mol­ecule of the title compound, C_16_H_23_N_3_O_5_, is twisted about the chiral C atom, the dihedral angle formed between the amide residues being 79.6 (3)°. The conformation about the imine bond [1.278 (5) Å] is *E*. In the crystal, O—H⋯O and N—H⋯O hydrogen bonding between the hy­droxy, amine and carbonyl groups leads to the formation of supra­molecular layers, which stack along the *c*-axis direction.

## Related literature

For background to the use of l-serine derivatives in anti-tumour therapy, see: Jiao *et al.* (2009 ▶); Yakura *et al.* (2007 ▶). For background to *N*-acyl­hydrazone derivatives from l-serine for anti-tumour testing, see: Pinheiro *et al.* (2010 ▶, 2011 ▶); de Souza *et al.* (2010 ▶); Howie *et al.* (2011 ▶).
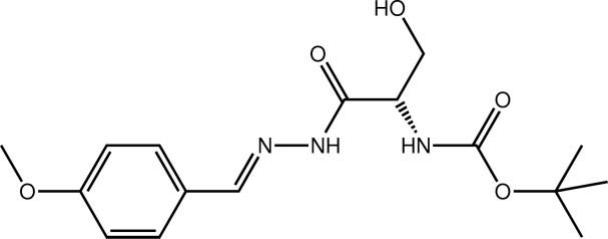


## Experimental

### 

#### Crystal data

C_16_H_23_N_3_O_5_
*M* *_r_* = 337.38
Triclinic, 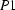

*a* = 5.3323 (4) Å
*b* = 5.7200 (4) Å
*c* = 14.3319 (10) Å
α = 79.919 (4)°
β = 83.686 (4)°
γ = 76.505 (4)°
*V* = 417.41 (5) Å^3^
*Z* = 1
Mo *K*α radiation
μ = 0.10 mm^−1^
*T* = 120 K
0.16 × 0.07 × 0.04 mm

#### Data collection

Bruker–Nonius Roper CCD camera on κ-goniostat diffractometer
Absorption correction: multi-scan (*SADABS*; Sheldrick, 2007 ▶) *T*_min_ = 0.887, *T*_max_ = 1.000
7495 measured reflections
1900 independent reflections
1661 reflections with *I* > 2σ(*I*)
*R*_int_ = 0.046

#### Refinement

*R*[*F* ^2^ > 2σ(*F* ^2^)] = 0.051
*wR*(*F* ^2^) = 0.113
*S* = 1.09
1900 reflections
230 parameters
6 restraints
H atoms treated by a mixture of independent and constrained refinement
Δρ_max_ = 0.24 e Å^−3^
Δρ_min_ = −0.25 e Å^−3^

### 

Data collection: *COLLECT* (Hooft, 1998 ▶); cell refinement: *DENZO* (Otwinowski & Minor, 1997 ▶) and *COLLECT*; data reduction: *DENZO* and *COLLECT*; program(s) used to solve structure: *SHELXS97* (Sheldrick, 2008 ▶); program(s) used to refine structure: *SHELXL97* (Sheldrick, 2008 ▶); molecular graphics: *ORTEP-3* (Farrugia, 1997 ▶) and *DIAMOND* (Brandenburg, 2006 ▶); software used to prepare material for publication: *publCIF* (Westrip, 2010 ▶).

## Supplementary Material

Crystal structure: contains datablock(s) global, I. DOI: 10.1107/S1600536811024263/hb5921sup1.cif

Structure factors: contains datablock(s) I. DOI: 10.1107/S1600536811024263/hb5921Isup2.hkl

Additional supplementary materials:  crystallographic information; 3D view; checkCIF report

## Figures and Tables

**Table 1:** Hydrogen-bond geometry (Å, °)

*D*—H⋯*A*	*D*—H	H⋯*A*	*D*⋯*A*	*D*—H⋯*A*
O3—H3o⋯O2^i^	0.84 (3)	1.87 (3)	2.651 (4)	153 (4)
N2—H2n⋯O3^ii^	0.88 (3)	1.93 (3)	2.803 (4)	169 (3)
N3—H3n⋯O5^iii^	0.88 (3)	2.34 (3)	3.188 (4)	164 (4)

Symmetry codes: (i) 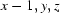; (ii) 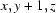; (iii) 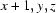.

**Fig. 1.**
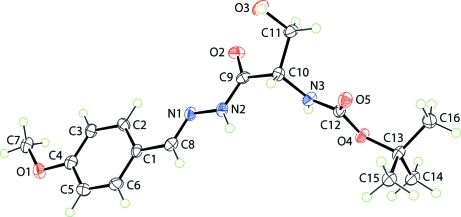
The molecular structure of (I) showing displacement ellipsoids at the 50% probability level.

**Fig. 2.**
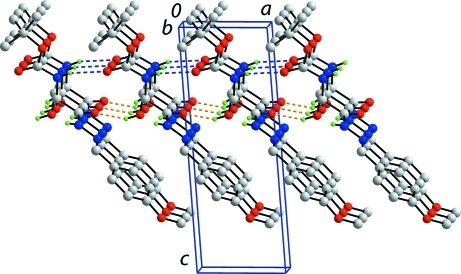
A view of the supramolecular array in the ab plane in (I) with the O—H···O and N—H···O hydrogen bonding shown as orange and blue dashed lines, respectively. Hydrogen atoms not participating in the hydrogen bonding scheme are omitted for reasons of clariy.

**Fig. 3.**
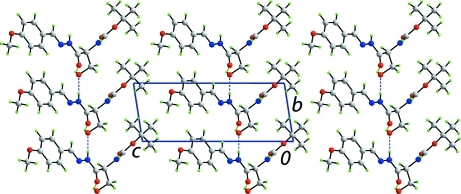
A view in projection down the a axis of the stacking of 2-D supramolecular arrays in the ab plane in (I), and with the O—H···O and N—H···O hydrogen bonding shown as orange and blue dashed lines, respectively.

## supplementary crystallographic information

### Comment

The anti-tumour activity of *L*-serine derivatives (Jiao *et al.*, 2009; Yakura *et al.*,2007) and the development of *N*-acylhydrazone derivatives from *L*-serine for use in anti-tumour testing (Pinheiro *et al.*, 2010; de Souza *et al.*, 2010; Pinheiro *et al.*, 2011: Howie *et al.*,2011) is well documented.

Although the absolute structure of (I), Fig. 1, could not be determined experimentally, the assignment of the *S*-configuration at the C10 atom is based on a starting reagent. The synthetic protocols led to the formation of both the *E* and *Z* isomers (see Experimental). Recrystallization provided one isomer only, with the conformation about the N1═C8 imine bond [1.278 (5) Å] being *E*. The molecule is twisted about the chiral centre as seen in the value of the N2—C9—C10—N3 torsion angle of 77.5 (4) °; the dihedral angle formed between the two amide residues, *i.e*. N2,C9,O2 and N3,C12,O5, is 79.6 (3) °. This arrangement precludes the formation of intramolecular hydrogen bonds. The methoxy residue is co-planar with the benzene ring to which it is attached as seen in the C7—O1—C4—C3 torsion angle of -0.5 (5) °.

The crystal packing is dominated by hydrogen bonding interactions whereby each of the acidic hydrogen atoms forms a hydrogen bond. Thus, the hydroxy-OH forms a hydrogen bond with the hydrazine-carbonyl, and at the same time accepts a hydrogen bond from the hydrazine-amine. The carbamate-amine forms a hydrogen bond with the carbamate-carbonyl; details are given in Table 1. The hydrogen bonding leads to layers in the *ab* plane, Fig. 2, which stack along the *c* axis, Fig. 3.

### Experimental

A reaction mixture of (*S*)-*t*-BuOCONHCH(CH_2_OH)CONHNH_2_ (1.0 mmol), prepared from *L*-serine (Howie *et al.*, 2011), and 4-methoxybenzaldehyde (1.05 mmol) in EtOH (10 ml) was refluxed for 4 h. The reaction mixture was rotary evaporated, and the residue was purified by washing with cold ethanol (3 *x* 10 ml): *M*.pt. 409 K, yield 80%. The solution NMR spectra in DMSO-d6 solution indicated the presence of both *E* and *Z* isomers. On recrystallization from EtOH for the structure determination, only the *E* isomer was obtained. ^1^H NMR (400 MHz, DMSO-d6): δ (p.p.m.): 11.28 and 11.21 (1*H*, s, NHN, *E* & *Z* isomers), 8.17 and 7.92 (1*H*, s, N=CH, *E* & *Z* isomers), 7.63 (1*H*, s, H1 or H5), 7.61 (1*H*, s, H1 or H5), 7.00 (2*H*, m, H2 and H4), 6.73 (d, J= 7.4) and 6.58 (d, J= 8.6), (1*H*, NHCH, *E* & *Z* isomers)), 4.91 (*m*) and 4.76 (t, J= 6.6), (1*H*, OH, *E* & *Z* isomers)), 4.91 and 4.02 (1*H*, m, CH, *E* & *Z* isomers)), 3.80 (3*H*, s, CH_3_O), 3.70–3.50 (2*H*, m, CH_2_OH), 1.39 (9*H*, s, (CH_3_)_3_C–). ^13^C NMR (100 MHz, DMSO-d6) δ (p.p.m.): 171.3 and 166.9 (COCH, *E* & *Z* isomers), 160.7 and 160.6 (C3, *E* & *Z* isomers), 155.2 (COO), 146.6 and 143.0 (N=CH, *E* & *Z* isomers), 128.5 and 128.3 (C1 and C5), 126.8 (C6), 114.3 (C2 and C4), 78.2 and 78.0 ((CH_3_)_3_C–, *E* & *Z* isomers)), 61.6 and 61.2 (CH_2_OH, *E* & *Z* isomers), 56.0 and 54.0 (CH, *E* & *Z* isomers), 55.3 (CH_3_O), 28.1 ((CH_3_)_3_C). IR (cm^-1^, KBr): 3306 (O—H), 1697 (COCH), 1678 (COO). EM/ESI: [M—H]: 336.1.

### Refinement

The C-bound H atoms were geometrically placed (C–H = 0.95–1.00 Å) and refined as riding with *U*_iso_(H) = 1.2–1.5*U*_eq_(C). The O– and N-bound H atoms were located from a difference map and refined with the distance restraints O–H = 0.84 ± 0.01 and N–H = 0.88±0.01 Å, and with *U*_iso_(H) = *zU*_eq_(carrier atom); *z* = 1.5 for O and *z* = 1.2 for N. In the absence of significant anomalous scattering effects, 1575 Friedel pairs were averaged in the final refinement. However, the absolute configuration was assigned on the basis of the chirality of the *L*-serine starting material.

### Crystal data

**Table:**

C_16_H_23_N_3_O_5_	*Z* = 1
*M**_r_* = 337.38	*F*(000) = 180
Triclinic, *P*1	*D*_x_ = 1.342 Mg m^−^^3^
Hall symbol: P 1	Mo *K*α radiation, λ = 0.71073 Å
*a* = 5.3323 (4) Å	Cell parameters from 14323 reflections
*b* = 5.7200 (4) Å	θ = 2.9–27.5°
*c* = 14.3319 (10) Å	µ = 0.10 mm^−^^1^
α = 79.919 (4)°	*T* = 120 K
β = 83.686 (4)°	Block, colourless
γ = 76.505 (4)°	0.16 × 0.07 × 0.04 mm
*V* = 417.41 (5) Å^3^	

### Data collection

**Table:**

Bruker–Nonius Roper CCD camera on κ-goniostat diffractometer	1900 independent reflections
Radiation source: Bruker-Nonius FR591 rotating anode	1661 reflections with *I* > 2σ(*I*)
graphite	*R*_int_ = 0.046
Detector resolution: 9.091 pixels mm^-1^	θ_max_ = 27.5°, θ_min_ = 3.7°
φ and ω scans	*h* = −6→6
Absorption correction: multi-scan (*SADABS*; Sheldrick, 2007)	*k* = −7→7
*T*_min_ = 0.887, *T*_max_ = 1.000	*l* = −18→18
7495 measured reflections	

### Refinement

**Table:**

Refinement on *F*^2^	Primary atom site location: structure-invariant direct methods
Least-squares matrix: full	Secondary atom site location: difference Fourier map
*R*[*F*^2^ > 2σ(*F*^2^)] = 0.051	Hydrogen site location: inferred from neighbouring sites
*wR*(*F*^2^) = 0.113	H atoms treated by a mixture of independent and constrained refinement
*S* = 1.09	*w* = 1/[σ^2^(*F*_o_^2^) + (0.0304*P*)^2^ + 0.3732*P*] where *P* = (*F*_o_^2^ + 2*F*_c_^2^)/3
1900 reflections	(Δ/σ)_max_ < 0.001
230 parameters	Δρ_max_ = 0.24 e Å^−^^3^
6 restraints	Δρ_min_ = −0.25 e Å^−^^3^

### Special details

**Table:**

Geometry. All s.u.'s (except the s.u. in the dihedral angle between two l.s. planes) are estimated using the full covariance matrix. The cell s.u.'s are taken into account individually in the estimation of s.u.'s in distances, angles and torsion angles; correlations between s.u.'s in cell parameters are only used when they are defined by crystal symmetry. An approximate (isotropic) treatment of cell s.u.'s is used for estimating s.u.'s involving l.s. planes.
Refinement. Refinement of *F*^2^ against ALL reflections. The weighted *R*-factor *wR* and goodness of fit *S* are based on *F*^2^, conventional *R*-factors *R* are based on *F*, with *F* set to zero for negative *F*^2^. The threshold expression of *F*^2^ > 2σ(*F*^2^) is used only for calculating *R*-factors(gt) *etc*. and is not relevant to the choice of reflections for refinement. *R*-factors based on *F*^2^ are statistically about twice as large as those based on *F*, and *R*- factors based on ALL data will be even larger.

### Fractional atomic coordinates and isotropic or equivalent isotropic displacement parameters (Å^2^)

**Table:**

	*x*	*y*	*z*	*U*_iso_*/*U*_eq_	
O1	1.6917 (5)	0.8513 (5)	0.7563 (2)	0.0280 (7)	
O2	0.9933 (5)	0.3081 (5)	0.3426 (2)	0.0259 (6)	
O3	0.4988 (5)	0.1450 (5)	0.3533 (2)	0.0244 (6)	
H3O	0.340 (3)	0.190 (9)	0.369 (3)	0.037*	
O4	0.4967 (5)	0.9626 (5)	0.06608 (19)	0.0228 (6)	
O5	0.2010 (5)	0.7831 (5)	0.1623 (2)	0.0250 (6)	
N1	1.0286 (6)	0.6618 (6)	0.4406 (2)	0.0215 (7)	
N2	0.8274 (6)	0.6827 (6)	0.3836 (2)	0.0209 (7)	
H2N	0.709 (6)	0.818 (5)	0.373 (3)	0.025*	
N3	0.6282 (6)	0.6919 (6)	0.1906 (2)	0.0212 (7)	
H3N	0.777 (5)	0.731 (8)	0.171 (3)	0.025*	
C1	1.2010 (7)	0.8412 (7)	0.5502 (3)	0.0194 (8)	
C2	1.4019 (7)	0.6394 (7)	0.5725 (3)	0.0211 (8)	
H2	1.4244	0.5028	0.5406	0.025*	
C3	1.5699 (7)	0.6361 (7)	0.6412 (3)	0.0222 (8)	
H3	1.7053	0.4975	0.6560	0.027*	
C4	1.5392 (7)	0.8349 (7)	0.6876 (3)	0.0229 (8)	
C5	1.3430 (8)	1.0405 (7)	0.6634 (3)	0.0256 (9)	
H5	1.3242	1.1793	0.6937	0.031*	
C6	1.1777 (8)	1.0429 (7)	0.5961 (3)	0.0251 (9)	
H6	1.0453	1.1836	0.5805	0.030*	
C7	1.8959 (8)	0.6473 (8)	0.7822 (3)	0.0280 (9)	
H7A	1.8249	0.5017	0.8027	0.042*	
H7B	1.9827	0.6773	0.8343	0.042*	
H7C	2.0207	0.6225	0.7274	0.042*	
C8	1.0108 (8)	0.8446 (7)	0.4829 (3)	0.0229 (8)	
H8	0.8738	0.9833	0.4709	0.027*	
C9	0.8245 (7)	0.4972 (7)	0.3388 (3)	0.0195 (7)	
C10	0.5925 (7)	0.5327 (7)	0.2797 (3)	0.0188 (7)	
H10	0.4336	0.6117	0.3161	0.023*	
C11	0.5605 (7)	0.2847 (7)	0.2643 (3)	0.0226 (8)	
H11A	0.4206	0.3063	0.2214	0.027*	
H11B	0.7227	0.1972	0.2339	0.027*	
C12	0.4209 (7)	0.8111 (7)	0.1416 (3)	0.0188 (7)	
C13	0.3072 (7)	1.1111 (7)	−0.0009 (3)	0.0213 (8)	
C14	0.4761 (8)	1.2529 (7)	−0.0707 (3)	0.0264 (9)	
H14A	0.5607	1.3426	−0.0360	0.040*	
H14B	0.3684	1.3678	−0.1172	0.040*	
H14C	0.6079	1.1395	−0.1037	0.040*	
C15	0.0964 (8)	1.2826 (7)	0.0503 (3)	0.0250 (8)	
H15A	−0.0152	1.1886	0.0920	0.038*	
H15B	−0.0070	1.4008	0.0034	0.038*	
H15C	0.1759	1.3688	0.0882	0.038*	
C16	0.1993 (8)	0.9468 (7)	−0.0514 (3)	0.0244 (8)	
H16A	0.3423	0.8281	−0.0770	0.037*	
H16B	0.0995	1.0457	−0.1036	0.037*	
H16C	0.0868	0.8610	−0.0063	0.037*	

### Atomic displacement parameters (Å^2^)

**Table:**

	*U*^11^	*U*^22^	*U*^33^	*U*^12^	*U*^13^	*U*^23^
O1	0.0280 (15)	0.0329 (16)	0.0243 (15)	−0.0048 (13)	−0.0056 (12)	−0.0074 (12)
O2	0.0193 (14)	0.0193 (13)	0.0391 (17)	−0.0037 (11)	−0.0067 (12)	−0.0026 (12)
O3	0.0160 (13)	0.0238 (14)	0.0286 (15)	−0.0031 (11)	−0.0011 (11)	0.0066 (11)
O4	0.0181 (13)	0.0237 (14)	0.0246 (14)	−0.0042 (11)	−0.0059 (11)	0.0041 (11)
O5	0.0229 (15)	0.0279 (15)	0.0241 (15)	−0.0076 (12)	−0.0041 (11)	0.0006 (12)
N1	0.0212 (16)	0.0245 (16)	0.0186 (16)	−0.0052 (13)	−0.0055 (12)	−0.0005 (13)
N2	0.0183 (16)	0.0231 (17)	0.0203 (17)	−0.0016 (13)	−0.0073 (13)	−0.0007 (13)
N3	0.0152 (15)	0.0221 (17)	0.0242 (17)	−0.0044 (12)	−0.0026 (13)	0.0034 (13)
C1	0.0201 (18)	0.0232 (19)	0.0155 (18)	−0.0087 (15)	−0.0010 (14)	0.0007 (14)
C2	0.0208 (19)	0.0228 (19)	0.0201 (19)	−0.0046 (16)	0.0018 (15)	−0.0072 (15)
C3	0.0189 (19)	0.026 (2)	0.0206 (19)	−0.0025 (15)	−0.0029 (15)	−0.0012 (16)
C4	0.0205 (19)	0.027 (2)	0.022 (2)	−0.0094 (16)	0.0011 (16)	0.0001 (16)
C5	0.028 (2)	0.0212 (19)	0.027 (2)	−0.0024 (16)	−0.0023 (17)	−0.0058 (16)
C6	0.026 (2)	0.0185 (18)	0.029 (2)	0.0007 (15)	−0.0069 (17)	−0.0021 (15)
C7	0.025 (2)	0.035 (2)	0.023 (2)	−0.0076 (18)	−0.0057 (16)	0.0015 (17)
C8	0.0228 (19)	0.0221 (19)	0.022 (2)	−0.0055 (15)	−0.0005 (15)	0.0016 (15)
C9	0.0176 (18)	0.0182 (17)	0.0209 (19)	−0.0059 (14)	0.0027 (14)	0.0020 (14)
C10	0.0157 (17)	0.0221 (18)	0.0180 (18)	−0.0044 (14)	−0.0016 (14)	−0.0008 (14)
C11	0.023 (2)	0.0199 (19)	0.025 (2)	−0.0073 (15)	−0.0042 (16)	0.0023 (15)
C12	0.0202 (19)	0.0202 (18)	0.0161 (18)	−0.0072 (14)	−0.0012 (14)	0.0001 (14)
C13	0.0178 (18)	0.0227 (19)	0.0209 (19)	−0.0029 (15)	−0.0051 (15)	0.0042 (15)
C14	0.027 (2)	0.022 (2)	0.027 (2)	−0.0028 (16)	−0.0056 (17)	0.0027 (16)
C15	0.027 (2)	0.022 (2)	0.024 (2)	−0.0027 (16)	−0.0046 (16)	−0.0024 (15)
C16	0.026 (2)	0.0255 (19)	0.022 (2)	−0.0068 (16)	−0.0025 (16)	−0.0036 (16)

### Geometric parameters (Å, °)

**Table:**

O1—C4	1.372 (5)	C5—H5	0.9500
O1—C7	1.425 (5)	C6—H6	0.9500
O2—C9	1.233 (5)	C7—H7A	0.9800
O3—C11	1.431 (4)	C7—H7B	0.9800
O3—H3O	0.841 (10)	C7—H7C	0.9800
O4—C12	1.350 (4)	C8—H8	0.9500
O4—C13	1.480 (4)	C9—C10	1.530 (5)
O5—C12	1.218 (4)	C10—C11	1.524 (5)
N1—C8	1.278 (5)	C10—H10	1.0000
N1—N2	1.389 (4)	C11—H11A	0.9900
N2—C9	1.336 (5)	C11—H11B	0.9900
N2—H2N	0.880 (10)	C13—C15	1.523 (5)
N3—C12	1.353 (5)	C13—C16	1.523 (5)
N3—C10	1.455 (5)	C13—C14	1.526 (5)
N3—H3N	0.880 (10)	C14—H14A	0.9800
C1—C2	1.397 (5)	C14—H14B	0.9800
C1—C6	1.401 (6)	C14—H14C	0.9800
C1—C8	1.469 (5)	C15—H15A	0.9800
C2—C3	1.397 (5)	C15—H15B	0.9800
C2—H2	0.9500	C15—H15C	0.9800
C3—C4	1.385 (5)	C16—H16A	0.9800
C3—H3	0.9500	C16—H16B	0.9800
C4—C5	1.400 (6)	C16—H16C	0.9800
C5—C6	1.372 (6)		
			
C4—O1—C7	117.4 (3)	N3—C10—C11	112.2 (3)
C11—O3—H3O	109 (3)	N3—C10—C9	109.8 (3)
C12—O4—C13	120.5 (3)	C11—C10—C9	109.1 (3)
C8—N1—N2	114.4 (3)	N3—C10—H10	108.6
C9—N2—N1	118.8 (3)	C11—C10—H10	108.6
C9—N2—H2N	119 (3)	C9—C10—H10	108.6
N1—N2—H2N	122 (3)	O3—C11—C10	110.0 (3)
C12—N3—C10	119.8 (3)	O3—C11—H11A	109.7
C12—N3—H3N	117 (3)	C10—C11—H11A	109.7
C10—N3—H3N	122 (3)	O3—C11—H11B	109.7
C2—C1—C6	118.3 (4)	C10—C11—H11B	109.7
C2—C1—C8	122.2 (3)	H11A—C11—H11B	108.2
C6—C1—C8	119.5 (3)	O5—C12—N3	124.9 (3)
C1—C2—C3	120.8 (3)	O5—C12—O4	125.6 (3)
C1—C2—H2	119.6	N3—C12—O4	109.5 (3)
C3—C2—H2	119.6	O4—C13—C15	110.6 (3)
C4—C3—C2	120.0 (3)	O4—C13—C16	110.2 (3)
C4—C3—H3	120.0	C15—C13—C16	112.6 (3)
C2—C3—H3	120.0	O4—C13—C14	101.7 (3)
O1—C4—C3	125.0 (3)	C15—C13—C14	110.9 (3)
O1—C4—C5	115.6 (3)	C16—C13—C14	110.4 (3)
C3—C4—C5	119.4 (3)	C13—C14—H14A	109.5
C6—C5—C4	120.4 (4)	C13—C14—H14B	109.5
C6—C5—H5	119.8	H14A—C14—H14B	109.5
C4—C5—H5	119.8	C13—C14—H14C	109.5
C5—C6—C1	121.0 (4)	H14A—C14—H14C	109.5
C5—C6—H6	119.5	H14B—C14—H14C	109.5
C1—C6—H6	119.5	C13—C15—H15A	109.5
O1—C7—H7A	109.5	C13—C15—H15B	109.5
O1—C7—H7B	109.5	H15A—C15—H15B	109.5
H7A—C7—H7B	109.5	C13—C15—H15C	109.5
O1—C7—H7C	109.5	H15A—C15—H15C	109.5
H7A—C7—H7C	109.5	H15B—C15—H15C	109.5
H7B—C7—H7C	109.5	C13—C16—H16A	109.5
N1—C8—C1	120.4 (3)	C13—C16—H16B	109.5
N1—C8—H8	119.8	H16A—C16—H16B	109.5
C1—C8—H8	119.8	C13—C16—H16C	109.5
O2—C9—N2	124.3 (4)	H16A—C16—H16C	109.5
O2—C9—C10	120.3 (3)	H16B—C16—H16C	109.5
N2—C9—C10	115.4 (3)		
			
C8—N1—N2—C9	−177.8 (3)	N1—N2—C9—C10	178.5 (3)
C6—C1—C2—C3	2.0 (6)	C12—N3—C10—C11	79.8 (4)
C8—C1—C2—C3	−176.0 (4)	C12—N3—C10—C9	−158.7 (3)
C1—C2—C3—C4	−0.3 (6)	O2—C9—C10—N3	−102.2 (4)
C7—O1—C4—C3	−0.5 (5)	N2—C9—C10—N3	77.5 (4)
C7—O1—C4—C5	−179.2 (4)	O2—C9—C10—C11	21.1 (5)
C2—C3—C4—O1	179.7 (4)	N2—C9—C10—C11	−159.2 (3)
C2—C3—C4—C5	−1.6 (6)	N3—C10—C11—O3	−173.9 (3)
O1—C4—C5—C6	−179.4 (4)	C9—C10—C11—O3	64.3 (4)
C3—C4—C5—C6	1.8 (6)	C10—N3—C12—O5	−5.9 (6)
C4—C5—C6—C1	−0.1 (6)	C10—N3—C12—O4	174.9 (3)
C2—C1—C6—C5	−1.8 (6)	C13—O4—C12—O5	−0.7 (5)
C8—C1—C6—C5	176.2 (4)	C13—O4—C12—N3	178.6 (3)
N2—N1—C8—C1	176.1 (3)	C12—O4—C13—C15	60.9 (4)
C2—C1—C8—N1	−1.8 (5)	C12—O4—C13—C16	−64.3 (4)
C6—C1—C8—N1	−179.8 (4)	C12—O4—C13—C14	178.7 (3)
N1—N2—C9—O2	−1.9 (5)		

### Hydrogen-bond geometry (Å, °)

**Table:**

*D*—H···*A*	*D*—H	H···*A*	*D*···*A*	*D*—H···*A*
O3—H3o···O2^i^	0.84 (3)	1.87 (3)	2.651 (4)	153 (4)
N2—H2n···O3^ii^	0.88 (3)	1.93 (3)	2.803 (4)	169 (3)
N3—H3n···O5^iii^	0.88 (3)	2.34 (3)	3.188 (4)	164 (4)

Symmetry codes: (i) *x*−1, *y*, *z*; (ii) *x*, *y*+1, *z*; (iii) *x*+1, *y*, *z*.
